# Emerging molecular therapies in the treatment of bladder cancer

**DOI:** 10.37349/etat.2024.00267

**Published:** 2024-08-29

**Authors:** Scott D. Bell, Anthony E. Quinn, Tom D. Spitzer, Brady B. Voss, Mark R. Wakefield, Yujiang Fang

**Affiliations:** IRCCS Istituto Romagnolo per lo Studio dei Tumori (IRST) “Dino Amadori”, Italy; ^1^Department of Microbiology, Immunology & Pathology, Des Moines University, West Des Moines, IA 50266, USA; ^2^Department of Surgery, University of Missouri School of Medicine, Columbia, MO 65212, USA; ^3^Ellis Fischel Cancer Center, University of Missouri School of Medicine, Columbia, MO 65212, USA

**Keywords:** Bladder cancer, molecular therapy, cancer therapy

## Abstract

Bladder cancer is a leading cancer type in men. The complexity of treatment in late-stage bladder cancer after systemic spread through the lymphatic system highlights the importance of modulating disease-free progression as early as possible in cancer staging. With current therapies relying on previous standards, such as platinum-based chemotherapeutics and immunomodulation with Bacillus Calmette-Guerin, researchers, and clinicians are looking for targeted therapies to stop bladder cancer at its source early in progression. A new era of molecular therapies that target specific features upregulated in bladder cancer cell lines is surfacing, which may be able to provide clinicians and patients with better control of disease progression. Here, we discuss multiple emerging therapies including immune checkpoint inhibitors of the programmed cell death protein 1 (PD-1)/programmed death ligand 1 (PD-L1) pathway, antibody-drug conjugates, modulation of the phosphoinositide 3-kinase (PI3K)/protein kinase B (AKT)/mammalian target of rapamycin (mTOR) cell proliferation pathway, chimeric antigen receptor T-cell therapy, and fibroblast growth factor receptor targeting. Together, these modern treatments provide potentially promising results for bladder cancer patients with the possibility of increasing remission and survival rates.

## Introduction

Bladder cancer (BC), commonly referred to as bladder urothelial carcinoma (UC), has been steadily rising in prevalence, accounting for an estimated 2.8% of all cancer deaths, and presents as a neoplastic invasion of the internal barrier urothelial cells and micturition-responsive smooth muscle that surrounds the bladder [1–3]. External risk factors for BC fall into several categories including smoking, diets, occupations, occupational agents, environmental factors, diseases, medications, and drugs. Smoking tobacco is largely recognized as one of the most significant risk factors in BC disease progression, with nearly 50% of BC cases attributed to smoking tobacco [4]. Occupations with an increased risk of BC include people working in aluminum production, rubber manufacturing, textile manufacturing, dry cleaning, the dye industry, painters, firefighters, and hairdressers. Environmental factors that show an increased risk of BC are arsenic compounds, X-ray and gamma-ray radiation, outdoor air pollution, and diesel exhaust. There are also genetic risk factors for BC. The heritability of BC was found to be approximately 30% in a study analyzing twins [5, 6]. The genetic risk of BC has been shown to be separate from external risk factors as well; thus, both are not needed to cause BC, and living an optimal lifestyle can reduce the risk of gaining BC across all unfavorable genetic loci [5].

A 0–IV system is followed when staging BC. Stage 0 is a non-invasive papillary carcinoma that has not spread to nearby lymph nodes, distant sites, organs, connective tissue, or muscle surrounding the bladder but has grown towards the hollow center of the bladder [7]. Stage I cancer indicates that the cancer has grown into the connective tissue surrounding the bladder but has not yet reached the muscle layer surrounding the bladder and there is still no spreading of the cancer into any local lymph nodes or distant sites [7]. Stage II BC has a split sub-staging of TIIA or TIIB. TIIA indicates that the cancer has spread into the inner layer of muscle, while TIIB indicates it has spread to the outer layer of muscle surrounding the bladder. In either case, the cancer has not yet spread into the layer of fatty tissue surrounding the bladder and there is still no spread to local lymph nodes or distant sites [7]. Stage III BC has a split sub-staging of TIIIA and TIIIB. Stage TIIIA can indicate the growth of the cancer through the muscle and into the fatty tissue surrounding the bladder and the possible spread of the cancer to either the prostate, seminal vesicles, uterus, or vagina. It has, however, not grown into the pelvic or abdominal wall at this stage. Stage TIIIB can indicate that the cancer has spread to two or more lymph nodes in the true pelvis or to lymph nodes along the common iliac arteries, but the cancer has still not spread to distant sites [7]. Stage IV is also split into stages TIVA and TIVB. Stage TIVA indicates that the cancer has grown through the bladder wall into the pelvic or abdominal wall, and the cancer has spread to distant lymph nodes. Stage TIVB indicates that the cancer has spread to distant organs [7].

Platinum-based chemotherapy is the standard first-line treatment for muscle-invasive BC preceding cystectomy. Cisplatin and carboplatin are commonly used platinum-based heavy metal alkylating agents capable of altering the DNA of metastatic cancer cells, thus inhibiting cancer progression. Cisplatin therapy has been optimized to the point that cisplatin treatment alone shows similar survival outcomes as surgical cystectomy of the bladder in some circumstances [8]. Carboplatin and cisplatin are shown to have similar outcomes in treating metastatic BC [9]. The significant drawbacks seen from the traditional chemotherapy treatment route are decreased quality of life in patients and patients being ineligible for further treatment due to diminished clinical conditions. Thus, clinicians are seeking targeted therapies that maintain patient quality of life.

Bacillus Calmette-Guerin (BCG) is the standard for treating non-muscle invasive BC. The mechanism of action for BCG is not fully understood, though it is believed to work through internalization by BC cells that lead to the activation of protective immune and inflammatory pathways. With BCG treatment, there is uncertainty due to the lack of information surrounding its mechanism of action, unsystematic administration scheduling, and adverse side effects.

**Figure 1:**
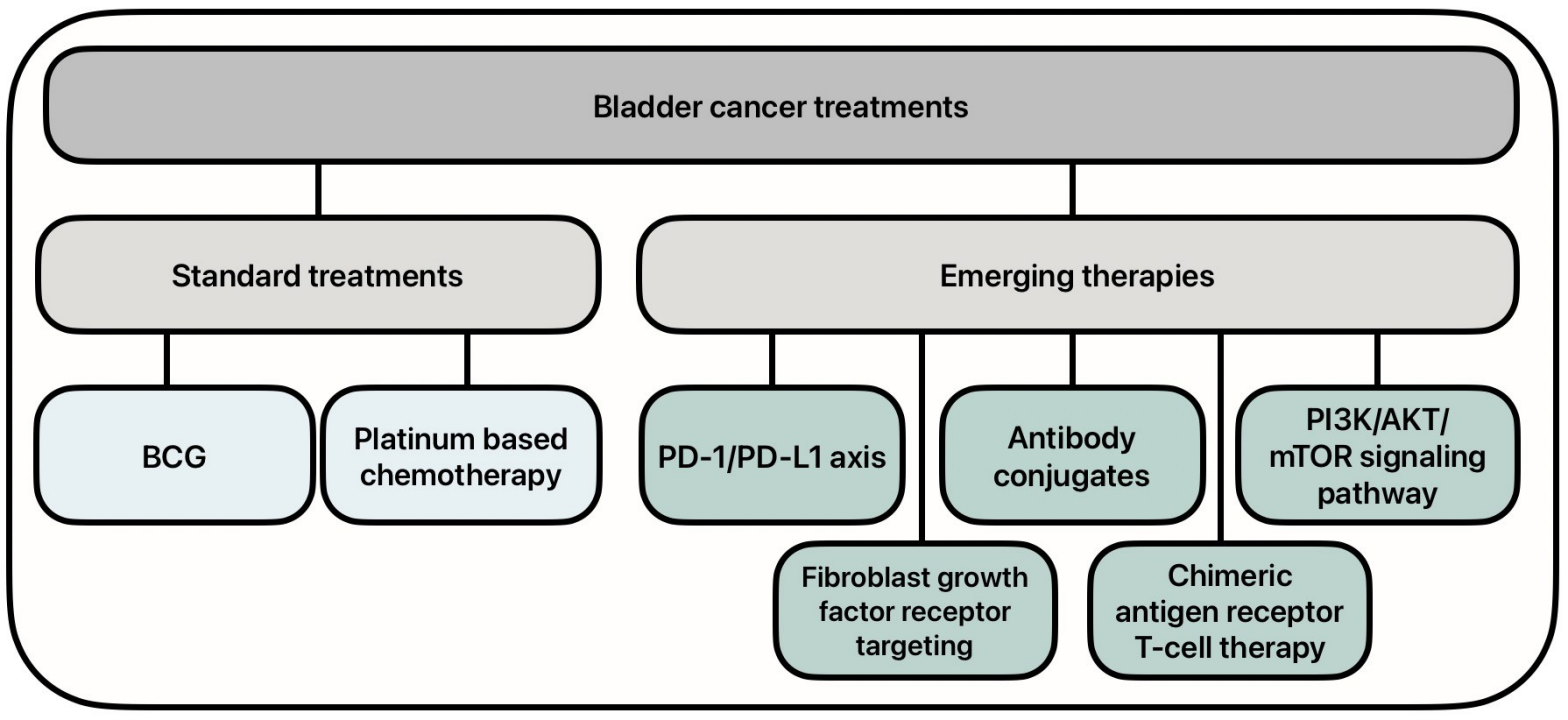
Current and emerging treatment options for bladder cancer. BCG: Bacillus Calmette-Guerin; PD-1/PD-L1: programmed cell death protein 1/programmed death ligand 1; PI3K/AKT/mTOR: phosphoinositide 3-kinase/protein kinase B/mammalian target of rapamycin

There has been an increased need for new therapies for the treatment of BC, as the standard treatments have shown to have decreased efficacy, especially in advanced disease states. Research has turned to molecular therapies to increase the efficacy of treatment. Emerging therapies include targeting the programmed cell death protein 1 (PD-1)/programmed death ligand 1 (PD-L1) axis and phosphoinositide 3-kinase (PI3K)/protein kinase B (AKT)/mammalian target of rapamycin (mTOR) (PAM) signaling pathways, as well as the use of antibody conjugates, fibroblast growth factor receptor (FGFR) inhibitors, and chimeric antigen receptor (CAR) T-cell therapy (Figure 1). These novel therapies have shown promise in the ongoing battle against BC.

## BCG therapy

BCG immunotherapy has been a part of the standard treatment of BC in patients for more than 30 years. Developed initially from re-cultured isolated colonies of *Mycobacterium bovis*, BCG was first utilized in tuberculosis prevention via vaccinations [10]. During that time, researchers studied the use of microbial products to treat cancer. The first successful use of BCG to treat patients with BC was reported by Morales et al. [11] in 1976, and the confirmation of its high efficacy propelled it to become a mainstay treatment of BCs [12]. Most patients with BC undergo complete resection accomplished via transurethral resection of bladder tumor with postoperative surveillance by cystoscopy followed by adjuvant treatment with intravesical BCG, reducing the risk of recurrence in patients [13, 14]. The original protocol for BCG is 120 mg BCG with 50 mL of saline administered via urethral catheter for 2 hours, repeated once per week for six weeks [11]. In current practice, treatments can range from 1 year to 3 years with up to 27 instillations. However, a minimum of 1-year treatment is required for BCG to be beneficial [15]. While the mechanism of action for BCG is not fully known, it is believed to work through BC cells’ internalization which leads to increased expression of major histocompatibility complex (MHC) class II, intercellular adhesion molecule I (ICAM-I), and pro-inflammatory cytokines, including IL-12 [12]. The combination of antigen presentation, increased cytokine levels, and dendritic cell upregulation leads to the recruitment of immune cells including granulocytes, CD4^+^ and CD8^+^ lymphocytes, natural killer (NK) cells, and macrophages [12]. NK cells have shown to be a key player in the BCG-mediated immune response, with BCG-specific NK cells being termed BCG-activated killer (BAK) cells [16]. In a mouse model study by Brandau and Böhle [16], it was found that NK-deficient mice were unable to elicit an effective BCG response. Additionally, it is believed that BAK cells work through the cytolytic protein perforin, which once released from granules, creates a pore in the target cells’ plasma membrane [17].

Different strains of BCG have been developed due to various labs re-culturing the mycobacterium [12]. The most studied strains in the last decade are Tice, Connaught, and RIVM, although Connaught was discontinued in 2018 [15, 18]. However, studies have not found a significant difference in overall survival (OS) with the different substrains [15].

While BCG has long been recognized as a standard of care for those with BC, studies have continued to try to find ways of optimizing immunotherapy to improve its efficacy. Recent and ongoing clinical studies are examining the potential effect of priming BC patients with the BCG vaccination before beginning intravesical BCG, hoping to improve its immune response. A recent mouse model study found that priming with BCG before instillation triggered a greater inflammatory process and accelerated T-cell entry into the bladder, compared to standard protocol [19]. S1602 is an ongoing phase III clinical trial that began in 2017, in which patients with non-muscular BC are primed or intradermally vaccinated with the Tokyo-172 BCG strain before being treated with intravesical therapies. The findings of the patients primed with the vaccination will be compared to those who have only been treated with the strain via intravesical treatment to see if there is any benefit to prior intradermal injections [20]. Although promising results have been reported in priming prior to intravesical BCG, more research is needed to fully understand the novel protocol’s immunotherapeutic abilities.

To further enhance the immunotherapy capabilities of BCG, research is currently being conducted in genetically modifying BCG to produce additional immunomodulators such as cytokines or chemokines. For example, Kanno et al. [21] enhanced BCG’s antitumor properties by genetically incorporating the detoxified S1 subunit of pertussis toxin into BCG, boosting the Th1 immune response in mice subjects. Although many studies have focused on recombinant BCGs, most have yet to advance to human clinical trials, except for strain VPM1002BC [22]. In a phase II study to test the efficacy of the strain, VPM1002BC showed that it has a recurrence-free rate of 49.3% at 60 weeks after trial registration and 43.7% after three years [23]. While these are promising results, further research is needed in recombinant BCGs to better demonstrate their efficacy.

## The PD-1/PD-L1 axis

**Figure 2:**
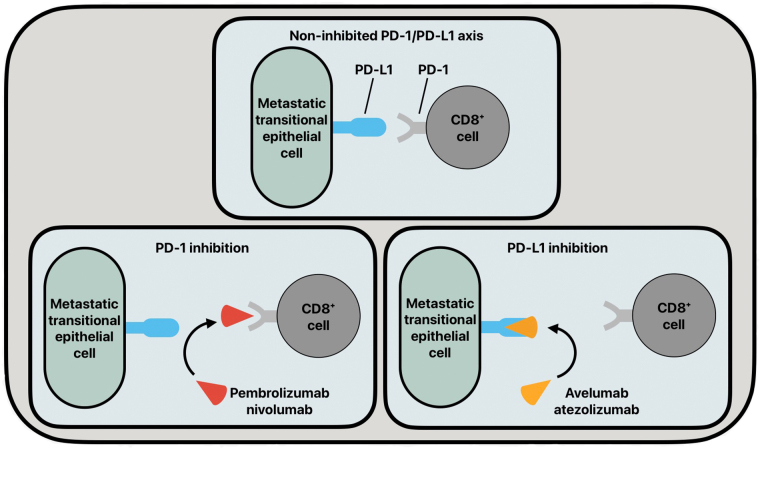
The common functioning of the PD-1/PD-L1 axis and its inhibition by various antibodies. PD-1: programmed cell death protein 1; PD-L1: programmed death ligand 1

PD-1 is a transmembrane glycoprotein expressed on the surface of cytotoxic CD8^+^ T-cells. Intraepithelial CD8^+^ T-cells are upregulated in UC pathologies and serve as a strong prognostic feature of UC [24]. PD-L1 is a transmembrane glycoprotein that is found to be upregulated in many tumorigenic cells. PD-L1 has been found to have increased expression in the urine of patients with non-muscle-invasive and muscle-invasive BC pathologies [25]. Further, it has been shown that the BC disease stage has a positive correlation with PD-L1 expression [26, 27]. The enhanced expression of PD-L1 in late-stage BC explains the increased interest in targeting the PD-1/PD-L1 axis in BC disease therapies. In cancer pathologies, CD8^+^ T-cells will have increased homing to tumorigenic cells, and in cell lines expressing increased PD-L1, such as BC cells, PD-L1 can bind to PD-1 on CD8^+^ T-cells to downregulate the immune response, and thus increase BC progression (Figure 2).

In recent years, monoclonal antibodies (mAbs) that target the PD-1/PD-L1 axis, such as pembrolizumab, nivolumab, avelumab, and atezolizumab, have been used in patient populations suffering from advanced-stage BC. These immune checkpoint inhibitors (ICIs) act as blockades to the progression of BC by inhibiting the PD-1/PD-L1 axis. Pembrolizumab and nivolumab are both immunoglobulin G4 (IgG4) mAbs targeted at PD-1. Pembrolizumab and nivolumab are nearly identical antibodies whose treatment efficacies differ in drug-independent differences [28]. Atezolizumab and avelumab are humanized IgG1 mAbs that target PD-L1 on metastatic transitional epithelial cells to inhibit the PD-1/PD-L1 axis. These inhibitory drugs show clinical promise, but their current drawback is that they are mainly being utilized under the scope of controlled clinical trials.

A recent study has shown that nivolumab increased disease-free survival in patients with high-risk muscle-invasive UC from 10.8 to 20.8 months [29]. Another group found that patients with advanced UC treated with pembrolizumab had an increased survival of 10.3 months versus a platinum-based chemotherapy treatment group, which survived an average of 7.4 months [30]. Another study found that when pembrolizumab was used in conjunction with enfortumab vedotin (EV), an antibody-drug conjugate (ADC) that targets the Nectin-4 protein on tumor cells, progression-free survival (PFS) was longer than traditional platinum-based chemotherapy treatment groups (Table 1) [31].

**Table 1:** Outcomes for trials involving the targeting of the PD-1/PD-L1 axis

**Clinical trial identification/reference**	**Clinical setting**	**Treatment setting**	**Outcome(s)**
NCT02632409/Bajorin et al. [29]	Patients with muscle-invasive urothelial carcinoma who had undergone radical surgery.	240 mg nivolumab or placebo was administered every 2 weeks for up to 1 year. Neoadjuvant cisplatin-based chemotherapy before trial entry was allowed.	Patients showed a median disease-free survival of 10.8 and 20.8 months when treated with placebo and nivolumab, respectively. Patients who were alive and free from recurrence outside the urothelial tract at 6 months were found to be 77.0% with nivolumab and 62.7% with placebo.
NCT02256436/Bellmunt et al. [30]	Patients with advanced urothelial cancer that recurred or progressed after platinum-based chemotherapy.	Patients were administered a dose of 200 mg pembrolizumab every 3 weeks or the investigator’s choice of chemotherapy with paclitaxel, docetaxel, or vinflunine.	The median overall survival in the total population was 10.3 months in the pembrolizumab group, as compared to 7.4 months in the chemotherapy group.
NCT04223856/Powles et al. [31]	Patients with unresectable locally advanced or metastatic urothelial carcinoma.	Patients were administered 3-week cycles of enfortumab vedotin (at a dose of 1.25 mg per kilogram of body weight intravenously on days 1 and 8) and pembrolizumab (at a dose of 200 mg intravenously on day 1) or gemcitabine and either cisplatin or carboplatin (determined on the basis of eligibility to receive cisplatin).	Progression-free survival was longer in the enfortumab vedotin-pembrolizumab group than in the chemotherapy group at a median of 12.5 months versus 6.3 months, respectively. Overall survival was longer in the enfortumab vedotin-pembrolizumab group than in the chemotherapy group at a median of 31.5 months versus 16.1 months, respectively.
NCT02387996/Sharma et al. [32]	Patients with metastatic or surgically unresectable locally advanced urothelial carcinoma.	Patients received nivolumab 3 mg/kg intravenously every 2 weeks until disease progression and clinical deterioration, unacceptable toxicity, or other protocol-defined reasons.	Overall survival was found to be a median 7.0 months.
NCT03891238/Iacovelli et al. [33]	Patients with metastatic urothelial carcinoma who were ineligible for cisplatin-based chemotherapy that were screened centrally for PD-L1 expression and only those with a tumour proportion score ≥ 5%.	Patients received 10 mg/kg avelumab intravenously every 2 weeks until disease progression, intolerable toxicity, or a decision to withdraw by the clinician/patient.	The median overall survival was 10.0 months and 43% of patients were alive at 1 year.
NCT02603432/Powles et al. [34]	Patients with unresectable locally advanced or metastatic urothelial cancer who did not have disease progression with first-line chemotherapy (four to six cycles of gemcitabine plus cisplatin or carboplatin) to receive best supportive care with or without maintenance avelumab.	Patients received either maintenance therapy with avelumab at a dose of 10 mg per kilogram of body weight, administered intravenously every 2 weeks plus best supportive care or best supportive care alone.	Overall survival at 1 year was 71.3% in the avelumab group and 58.4% in the control group. Median overall survival was 21.4 months in the avelumab group and 14.3 months in the control group.
NCT04624399/Castellano et al. [35]	Patients were in disease stages prior to cystectomy in non-urothelial muscle-invasive bladder cancer	Patients received 2 cycles of atezolizumab 1200 mg once every three weeks prior to cystectomy.	The pathological complete response rate in pT2 or above patients was 38% (8/21), and 35% (8/23) including pT1 patients.

PD-1/PD-L1: programmed cell death protein 1/programmed death ligand 1

Nivolumab is currently being used mainly as a resource for patients with progressive late-stage UC. A study found that in patients with high-risk muscle-invasive UC who had undergone radical surgery, there was an increase in disease-free survival time compared to placebo groups [29]. A multi-center, phase II, single-arm study using nivolumab in patients who had received platinum-based chemotherapy for metastatic UC showed that nivolumab provided a clinical improvement to the post-chemotherapy treatment group (Table 1) [32].

Avelumab and atezolizumab are relatively new antibody therapies targeting PD-L1 in metastatic urothelial cells. In cisplatin chemotherapy ineligible patient groups, avelumab has been shown to be a viable alternative therapy for the OS of patients with metastatic UC (Table 1) [33]. Avelumab has significantly increased the survival time of patients receiving avelumab alongside supportive care versus those only receiving supportive care (Table 1) [34]. In early clinical trials, atezolizumab has been shown to be a safe neoadjuvant therapy for the treatment of muscle-invasive BC (Table 1) [35].

## Antibody-drug conjugates

**Figure 3:**
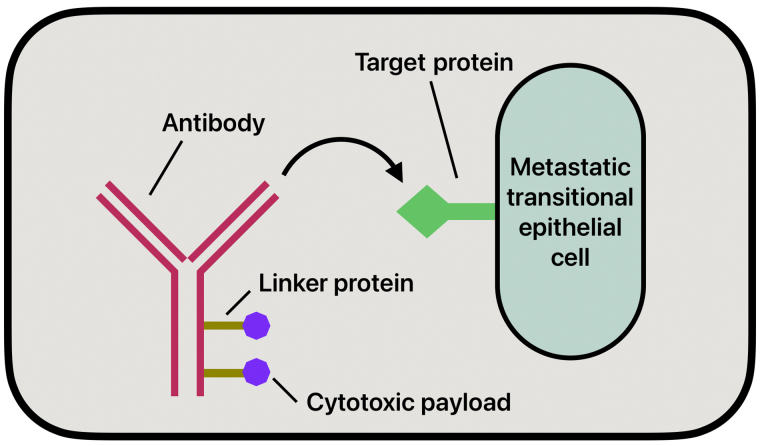
Antibody-drug conjugate simplified mechanism of action

ADCs have emerged as a new therapeutic option in treating BC, especially in advanced stages or when the tumor becomes refractory to standard treatment methods. The mechanism of action of ADCs works by targeting antigens overly expressed on the surface of tumor cells, attaching to them, and allowing for the selective delivery of cytotoxic agents directly to the tumor (Figure 3). The aim of this therapy is to deliver targeted drugs directly to the metastatic cells while minimizing systemic toxicity. This direct delivery of drugs allows for a reduction of damage to healthy tissues and greater efficacy in treatment [36]. The three components that comprise an ADC are mAbs, linkers, and the cytotoxic payload.

ADCs are mAbs covalently linked to small molecule anticancer agents, specifically designed to target antigens overexpressed on the surface of tumor cells [36]. The most expressed antigens in BC include HER-2, TROP-2, and Nectin-4 [36]. However, unlike traditional mAbs, the antibodies in ADCs are engineered to deliver a cytotoxic payload without eliciting an immune response. The antibody is chosen based on the identification of an antigen strongly expressed by malignant cells that is also absent in nonmalignant cells to reduce overall systemic toxicities [37] Commonly used mAbs include IgG, particularly subclass IgG1. IgG1 is often used due to its low molecular weight, long half-life, and ability to stimulate immune effector functions [36].

The linker component of an ADCs functions as a conjugate between the antibody and cytotoxic drug through the antibody’s heavy chain [36]. The two major classes of linkers are cleavable and non-cleavable linkers. Cleaver linkers release the cytotoxic payload from the mAb in the presence of a tumor microenvironment through various mechanisms. Some of these mechanisms include hydrolysis facilitated by low endosomal or lysosomal pH, lysosomal protease activity, or disulfide bond breakage mediated by high intracellular concentrations of glutathione in tumor cells [38]. Each mechanism is associated with different ADCs. For example, ADCs indatuximab ravtansine and mirvetuximab soravtansine are glutathione-sensitive linkers, and sacituzumab govitecan (SG) and EV are protease-dependent linkers [39]. Non-cleavable linkers are more stable and depend on degrading the entire antibody-linker complex to release the payload. Examples include trastuzumab emtansine (T-DM1) and belantamab mafodotin [40].

The payloads in ADCs are highly potent cytotoxic drugs that, due to their toxicity, cannot be used as standalone treatments. Payload types include microtubule inhibitors, like auristatins and maytansins, DNA damaging agents, like calicheamicins and duocarmycins, and other toxin types [36].

EV is an ADC composed of the human IgG1 monoclonal antibody that is conjugated to cytotoxic agent monomethyl auristatin E (MMAE) via a protease-cleavable linker. It specifically targets Nectin-4, a cell adhesion antigen overexpressed in BC. The clinical efficacy of EV was first studied through a phase I trial, EV-101, which evaluated the ADC in patients with locally advanced or metastatic BC who had undergone extensive and various prior treatments. Study participants received EV in escalating doses up to 1.25 mg/kg on days 1, 8, and 15 of a 28-day cycle. Among the 112 patients with metastatic BC who received EV as a single-agent therapy at a dose of 1.25 mg/kg, the objective response rate (ORR) was 43%, with the duration of response averaging 7.4 months. The study found that the median OS was 2.3 months, with a one-year OS rate of 51.8 (Table 2) [41].

**Table 2:** Outcomes for trials involving the use of antibody-drug conjugates

**Clinical trial identification/reference**	**Clinical setting**	**Treatment setting**	**Outcome(s)**
CT02091999/Rosenberg et al. [41]	Patients with Nectin-4-expressing solid tumors who progressed on ≥ 1 prior chemotherapy regimen and/or programmed death-1 (PD1) receptor/programmed death ligand-1 (PDL-1) inhibitor, including a cohort of patients with metastatic urothelial carcinoma who received prior anti-PD-L1 therapy.	Patients received escalating doses of enfortumab vedotin up to 1.25 mg/kg on days 1, 8, and 15 of every 28-day cycle.	Median overall survival was 12.3 months, and the overall survival rate at 1 year was 51.8%.
NCT03219333/Yu et al. [42]	Patients with locally advanced or metastatic urothelial carcinoma previously treated with PD-1 or PD-L1 inhibitors. Cohort 2 included adults (aged ≥ 18 years) with an Eastern Cooperative Oncology Group performance status score of 2 or less who were considered ineligible for cisplatin at enrolment and who had not received platinum-containing chemotherapy in the locally advanced or metastatic setting.	Enfortumab vedotin was given intravenously at a dose of 1.25 mg/kg on days 1, 8, and 15 of every 28-day cycle.	Objective response rate was 52% with 18 of 89 patients achieving a complete response and 31% of patients achieving a partial response.
NCT03474107/Rosenberg et al. [43]	Patients with locally advanced or metastatic urothelial carcinoma who had received prior platinum-containing chemotherapy and had disease progression during or after PD-1/L1 inhibitor treatment.	Patients randomized to enfortumab vedotin received doses of 1.25 mg/kg (maximum weight, 100 kg) on days 1, 8, and 15 of each 28-day cycle and those randomized to chemotherapy received docetaxel 75 mg/m^2^, paclitaxel 175 mg/m^2^, or vinflunine 320 mg/m^2^ (capped at 35% of patients) on day 1 of each 21-day cycle.	Risk of death was reduced by 30% with enfortumab vedotin versus chemotherapy. Median overall survival estimates were 12.91 months and 8.94 months for enfortumab vedotin and chemotherapy, respectively.
NCT03547973/Tagawa et al. [44]	Patients with locally advanced or unresectable or metastatic urothelial carcinoma who had progressed after prior platinum-based combination chemotherapy and checkpoint inhibitors.	Patients received sacituzumab govitecan 10 mg/kg on days 1 and 8 of 21-day cycles.	At a median follow-up of 9.1 months, the objective response rate was 27%; 77% had a decrease in measurable disease. The median duration of response was 7.2, with median progression-free survival and overall survival of 5.4 months and 10.9 months, respectively.

Phase II and phase III trials were conducted based on the promising results of the phase I trial, EV-101. The EV-201 phase II trial included participants with locally advanced or metastatic BC and were divided into two cohorts. The participants that made up cohort 1 were those who were refractory to both platinum-based and anti-PD-1/PD-L1 therapy. Cohort 2 was made up of participants with previous anti-PD-1/PD-L1 therapy but who were never treated with platinum chemotherapy. In cohort 1, the ORR was 44%, with a complete response (CR) rate of 12% and a median duration of response (mDOR) of 7.6 months. Cohort 2 demonstrated an ORR of 52%, a 20% CR rate, and an mDOR of 10.9 months (Table 2) [42].

The phase III EV-301 trial compared EV to chemotherapy in locally advanced or metastatic BC patients resistant to both platinum-based and anti-PD-1/PD-L1 therapy. 301 patients received EV, and it was found that their median OS was extended by four months, and the median PFS improved by 1.8 months, compared to the 307 patients undergoing chemotherapy. The ORR with EV treatment was significantly higher at 41%, compared to chemotherapy at 18%. These findings led to FDA approval of use of EV in treatment for locally advanced or metastatic BC patients who showed refractory results to platinum-based and anti-PD-1/PD-L1 therapies (Table 2) [43].

SG is an ADC that targets TROP-2, a transmembrane glycoprotein overexpressed in many epithelial malignancies, including BC. SG is conjugated to SN-38, an active metabolite of irinotecan that inhibits topoisomerase I to prevent DNA replication and transcription. Preclinical studies and early human trials of SG across different epithelial cancers showed some antitumor activity [45]. The TROPHY-U-01 study assessed SG in patients with muscular BC refractory to platinum chemotherapy and PD-1/PD-L1 checkpoint inhibitor treatment. The study’s results showed an ORR of 27%, with a median PFS of 5.4 months and an OS of 10.9 months (Table 2) [44]. Based on the study’s outcomes, the FDA granted accelerated approval to SG for patients with muscular BC who received and demonstrated refractory responses to both platinum-containing chemotherapy and PD-1/PD-L1 inhibitor therapy [46].

Human epidermal growth factor receptor 2 (HER2) is an overexpressed antigen in various cancers, including breast and BCs. ADCs targeting the HER2 antigen, like T-DM1, have been approved for treating breast cancer, and its efficacy in the treatment of BC has also been studied; however, with mixed results [47]. Disitamab vedotin, another ADC, connects a HER2-targeting antibody to MMAE, and its efficacy is currently being studied in both gastric and BCs [48]. Clinical trials have demonstrated ADC’s effectiveness in treating HER2-positive BC, with promising response rates and survival outcomes [48].

While much is not currently known about the resistance of ADCs in the treatment of BC, there is some evidence that ADC resistance can occur through various mechanisms, including lack of antigen attachment, and changes in the cell cycle [49]. A common cause to reduced efficacy of ADC is loss of target antigen. This can occur due to gene mutations leading to antigen concealment by the immune system, downregulation of the target gene expression, or selection of tumor cell clones with lower levels of target antigen expression [49]. The loss of the target antigen can lead to a reduction of antibody binding and release of payload. A study on patients with metastatic triple-negative breast cancer (TNBC) demonstrated that a loss of Trop-2 expression was linked to a reduced response to SG treatment [50]. Furthermore, The ASCENT trial found that patients with metastatic TNBC with high Trop-2 expression treated with SG experienced better outcomes compared to those with low or absent Trop-2 expression [51]. Research is currently being conducted to see if loss of the target antigen can be mediated by bispecific antibodies, which are antibodies that are able to target multiple antigens. Cell cycle alterations can also lead to ADC resistance, as the cell cycle plays a role in tumorigenesis, as well as establishing novel resistance mechanisms [40]. In a preclinical trial, Sabbaghi et al. [52] found significant increases in cyclin B expression in T-DM1-resistant cells. Additionally, alterations in the apoptosis pathway may affect the effectiveness of ADCs. There is evidence of overexpression and mutation of BCL-X and BCL-2, as well as impaired regulation of the BAX and BAK proteins, in patients treated with gemtuzumab ozogamicin [53]. Other evidential mechanisms of ADC resistance include suppression of payload efficacy, impairment of vesicle pathways, and the tumor microenvironment itself [49].

While ADC resistance is a current challenge, advances in ADC engineering are continually addressing these obstacles. Novel cleavable linkers with membrane-permeable payloads improve efficacy against target-negative cells, expanding treatment to cancers with low target expression [54]. In preclinical trials, the use of bispecific and biparatopic antibodies have shown to be promising, as they can recognize two different antigens on the same antigen or bind two non-overlapping epitopes of the same antigen, respectively [49]. Combining ADCs with ICIs has also shown promise in overcoming resistance. ICIs enhance the body’s immune response, while ADCs use antibodies to deliver cytotoxic drugs [55]. These two therapeutics provide synergistic effects that may improve outcomes in resistant tumors.

The utilization of ADCs in treating BC has allowed for the delivery of potent cytotoxic agents to directly target tumor cells while minimizing its effect on the immune system. Early results are promising, such as those from EV-103, that have showed a high ORR when combined with pembrolizumab [36–38]. Ongoing trials aim to enhance efficacy, understand resistance mechanisms, and reduce toxicities while continuing to explore ADC’s therapeutic abilities in treating BC [40].

## The PI3K/AKT/mTOR signaling pathway

**Figure 4:**
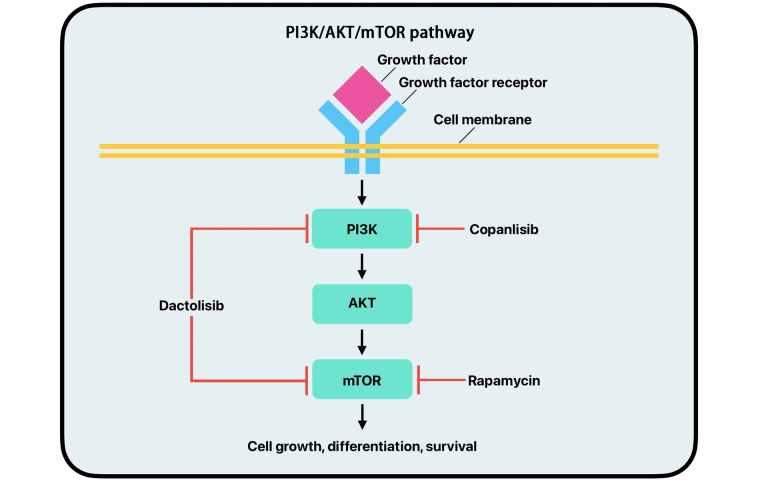
Simplified mechanism of action of the PI3K/AKT/mTOR pathway with copanlisib, dactolisib, and rapamycin drug inhibition. PI3K: phosphoinositide 3-kinase; AKT: protein kinase B; mTOR: mammalian target of rapamycin

The PAM pathway is a signal transduction network that plays a major role in cell growth, survival, and division (Figure 4). Abnormal activity of the PAM pathway is associated with many hallmarks of cancer, including cell proliferation, autophagy, apoptosis, angiogenesis, epithelial-to-mesenchymal transition, and chemoresistance [56]. PI3K is a group of kinases; the most significant byproduct of kinase action in relation to the PAM pathway is PIP3. PIP3 allows for the recruitment of AKT and PDK-1 to proteins that act as mediators in the PI3K pathway. PIP3 also facilitates the phosphorylation and activation of AKT by PI3K. The activation of AKT determines the regulation of many proteins downstream of it that promote protein synthesis, cell growth, cell survival, and motility. Some of the most important proteins regulated by AKT are mTOR proteins [57]. The mTOR proteins are regulators of cell growth, metabolism, cell division, and survival, and the AKT protein regulates these proteins in response to environmental stressors. Mutations to genes that control the PAM pathway are prevalent in cancerous cells. Dysregulation of this pathway can lead to tumor progression and initiation [56, 58–60].

Dysregulation of the PAM pathway can lead to unregulated cell growth, division, metabolism, and protein synthesis contributing to cancer. New studies focus on specific parts of the PAM pathway rather than total inhibition [60]. PI3K inhibitors work to inhibit different kinases along the PI3K pathway [61]. Several of these inhibitors have been assessed for efficacy in clinical trials. Copanlisib, alpelisib, buparlisib, and pilaralisib are PI3K inhibitors that have been featured in multiple clinical trials (Figure 4). These trials have shown moderate efficacy and substantial toxicity [60, 62–64]. Many of these clinical trials have occurred in breast cancer and leukemia; however, more studies have been done on PI3K inhibitors for BC in recent years, which have shown promising results [65, 66].

PI3K inhibitor resistance is a pressing concern, and evidence shows that it is often due to factors like mutations and amplification of PI3K, drug-related toxicities, feedback upregulation leading to compensatory mechanisms, non-coding RNA in regulating PI3K signaling, or enhanced insulin production upon PI3K inhibition [67]. Furthermore, PI3K inhibitors show ranging efficacies in different populations, showcasing a need for proper patient selection [68]. However, recent preclinical and phase I studies are focusing on novel PI3K inhibitors that aim to reduce their resistance [67].

AKT inhibitors have been used therapeutically for several other cancers due to AKT’s significance in activating mTOR, specifically mTOR complex 1 (mTORC1), which plays a significant role in cell metabolism and anabolism [60, 69]. AKT inhibitors work by interfering with the activation of AKT thereby inhibiting mTORC1 and the subsequent angiogenesis, autophagy, apoptosis, cell proliferation, and chemoresistance [56]. AKT inhibitors fit into six different classes. The first class works by inhibiting AKT1 and AKT2. The second class works to prevent the generation of PIP3 by PI3K via lipid-based AKT inhibitors [70]. The third class consists of pseudosubstrate inhibitors, inhibiting PKC catalytic activity [70, 71]. The fourth class consists of allosteric inhibitors of the AKT kinase domain. The fifth class consists of antibodies. The sixth class of AKT inhibitors includes compounds that interact with the pleckstrin homology domain of AKT [35]. The AKT inhibitor, ATP-competitive pan-AKT, has been studied in several trials and shows promising results [60, 64, 72, 73]. A certain type of AKT inhibitors, allosteric pan-AKT inhibitors, are in early development and have shown to be potent and precise in AKT inhibition while providing relatively lower toxicity than other inhibitors [60, 74, 75].

The mechanisms underlying acquired resistance to AKT inhibitors have not undergone thorough investigation. However, evidence shows that elevations in certain biomarkers can be predictive of resistance [76]. Also, newer studies have demonstrated that combining PI3/AKT inhibitors with other therapeutics, like epidermal growth factor receptor (EGFR) inhibitors, may reduce resistance to AKT inhibitors [77].

While AKT and PI3K inhibitors are relatively new treatments, mTOR inhibitors have been used clinically for the past decade. Due to the significance of mTOR in the cell life cycle, it has been heavily studied, and many mTOR inhibitors have been approved for clinical use. The first approved form of mTOR inhibitors was rapamycin [60, 78–81]. Rapamycin works by directly inhibiting mTOR (Figure 4). However, it is not used due to its poor aqueous solubility and chemical stability [82]. The first generation of mTOR inhibitors were all rapamycin analogs, which are all FDA-approved and have shown positive results in BCs. However, they have all shown significant side effects in phase II clinical trials due to toxicity [60, 83–86]. Second-generation mTOR inhibitors act by binding directly to the ATP site of mTOR to exhibit inhibitory effects and are still in the early stages of clinical trials [60, 82, 87].

Resistance to mTOR inhibitors has been shown to have several mechanisms. Some of these include mutations in mTOR itself and activation of the PI3K/AKT pathway, both diminishing drug efficacy. Additionally, increased extracellular regulated kinase/microtubule-associated protein kinase (ERK/MAPK) pathway signaling can contribute to resistance by enhancing mTORC1 function. However, combining mTOR inhibitors with agents that target these pathways could potentially improve therapeutic outcomes [88].

The mTOR pathway regulates cell proliferation, survival, and metabolism. It can also be important in tumor initiation, progression, therapy resistance, and outcomes. It has been shown that UC alters various portions of this pathway, leading to a poorer prognosis. mTORC1 is a master regulator of cellular anabolism and promotes cell metabolism, growth, and proliferation. Upregulation of mRNA translation and protein synthesis is another primary function of mTORC1. Two important downstream targets are eukaryotic translation initiation 4E-binding protein 1 (4EBP1) and ribosomal protein S6 kinase 1 (S6K1). When mTORC1 is activated, it phosphorylates 4EBP1, interrupting its ability to form a complex with eIF4A, allowing for cell cycle progression. The upregulation of mTORC1 allows cancer cells to increase in size and number during tumorigenesis. While mTOR inhibitors are not new, second-generation inhibitors are relatively novel. These inhibitors include ATP-competitive mTOR inhibitors and PI3K/mTOR dual inhibitors. ATP-competitive mTOR inhibitors are highly selective to mTOR and can inhibit mTORC1 and mTORC2, thus decreasing cell proliferation. Several PI3K/mTOR dual inhibitors have shown promise in preclinical and early clinical trials. Dactolisib, a PI3K/mTOR dual inhibitor, has been shown to suppress tumor cell growth by inducing cell cycle arrest and caspase-dependent apoptosis in eight different human BC cell lines (Figure 4). However, further research must examine dual-drug treatment approaches and ways of overcoming treatment resistance. Combining medications that can aid the mTOR inhibitors in overcoming the treatment resistances would open new approaches to treating UC [59].

## CAR-T cell therapy

CAR is an artificial membrane protein composed of three domains: an extracellular domain, a transmembrane domain, and an intracellular domain [89]. The extracellular domain is a single-chain variable fragment (ScFv) that can bind tumor-associated antigens (TAA) without MHC restriction [90], while the transmembrane domain consists of CD8, stabilizing CAR, and the intracellular domain mediates T-cell activation [91, 92]. During therapy, CAR-T cells enter tumor tissue, causing ScFv fragments to bind their antigens. This elicits an activation signal from the transmembrane domain to the intracellular domain, activating T-cells to kill the tumor cells [93]. T-cells for CAR-T cell therapy are taken from the patient’s peripheral blood, infected with a virus containing the CAR plasmid, and reinjected into the patient [93].

CAR-T cell therapy requires a molecular target, typically TAAs, to allow T-cells to target tumor cells selectively. Fortunately, there is no shortage of targets in BC cells, and studies have shown that specific markers, including PD1, MUC1, and EGFR, are highly expressed in BC cancer cells and can serve as potential therapeutic targets [94].

In a preclinical study, Parriott et al. [95] developed chimeric PD-1 (chPD1) receptors toward PD-1, a receptor expressed on the surface of tumor cells in many cancers, including BC. They found that chPD1 T-cells showed a significant increase in proinflammatory cytokine secretion, leading to the lyses of PD-1-expressing tumor cells. Additionally, compared to naive mice, mice challenged with the previously rejected tumor type had 100% rejection and long-term tumor-free survival.

Other studies have been used as a proof of concept for CAR-T cell’s ability to target specific receptors or enhance overall effectiveness. In a study by Yu et al. [96], the group successfully targeted MUC1 on patient BC organoids using CAR-T cells and confirmed its ability to elicit an immune response. In an additional study, Grunewald et al. [97] demonstrated that epigenetic modification of BC cells could enhance EGFR-specific CAR-T cell effectiveness. In the study, EGFR CAR-T cells paired with the hypomethylation drug, decitabine, significantly increased CAR-T cells’ overall BC cell killing. Decitabine increased CAR-T cell-killing ability by altering gene expression to favor apoptosis, leading to a more effective treatment [97].

Some potential draw backs of CAR-T cell therapy are that there are few clinical trials supporting its effectiveness in BC. Additionally, it has been found that many cancers express extracellular *N*-glycan, which, in high concentrations, can significantly impact the effectiveness of CAR-T cell activity [98]. However, other studies have shown that in mice models, knocking out glycosylation genes can improve therapy, providing a potential solution [99]. With emerging research, CAR-T cells show promise in the treatment of BC.

## Fibroblast growth factor receptor (FGFR) targeting therapies

FGFR targeting therapies are among the more newly available therapies for BC. The FGFR pathway is initiated by the expression of FGFR genes, producing a subgroup of receptor tyrosine kinases called FGFRs found on the surface of cells [100]. When bound to FGF molecules, FGFRs trigger signals to induce certain cell functions. FGFR1–4 plays crucial roles in cell proliferation, growth, and other cellular functions. Mutations in these receptor genes can activate pathways such as PI3K/AKT, which can play significant roles in oncogenesis and proliferation [101]. Approximately 20% of advanced BC cases are estimated to have FGFR3 mutations [102]. The high prevalence of these mutations has led to the development of FGFR pathway inhibition therapeutics.

Erdafitinib is the first and only FGFR inhibitor approved by the FDA to treat BC patients with FGFR2 and FGFR3 mutations who previously had platinum-based chemotherapy [101]. In a phase I study, 189 patients with advanced solid tumors from various primary sites, including the breast, liver, and bladder, were treated with Erdafitinib. Patients with bladder tumors had the most significant responses, with the ORR shown to be 46% in patients with BC who had an FGFR mutation [103]. These promising results led to the BLC2001 Phase II study. In this study, erdafitinib was administered to 99 patients with advanced or metastatic BC with FGFR gene mutations. Doses of erdafitinib were given 8 mg daily for 14 days and increased to 9 mg after if no side effects were observed. The ORR was 40%, with 3% having a CR and 37% having a partial response. Among patients with FGFR3 mutations, 36 of 74 responded to treatment, and 4 of 25 with FGFR2/3 fusions responded. The mDOR was 5.6 months, the median PFS was 5.5 months, and the median OS was 13.8 months [104]. The results of the BLC2001 trial led to erdafitinib FDA approval and more studies, some of which have compared erdafitinib to chemotherapy and tested its efficacy with immunotherapy.

The phase III THOR trial compared erdafitinib to chemotherapies, either docetaxel or vinflunine, in patients with BC with FGFR2/3 previously treated with other therapies. Results showed that erdafitinib had a median OS of 12.1 months compared to 7.8 months for chemotherapy. The median PFS was also higher in erdafitinib than chemotherapy, with results showing 5.6 months and 2.7 months, respectively. The ORR was 46% for erdafitinib compared to 12% for chemotherapy [105]. Demonstrating its effectiveness, erdafitinib received full approval for treating platinum-refractory metastatic BC.

The phase II new-onset refractory status epilepticus (NORSE) trial compared erdafitinib alone and in combination with cetrelimab, a PD-1 monoclonal antibody in patients with metastatic BC not previously treated with cisplatin-based chemotherapy. The study found that the ORR of erdafitinib was 44.2%, with one CR, and 54.5% for the combination therapy, with six CR. The median PFS was also longer with the combination therapy at 10.97 months compared to erdafitinib alone at 5.62 months [106]. While cetrelimab is not FDA-approved for use in any cancer, the trial showed promising results for the combination therapy in difficult to treat BC.

Adverse side effects of FGFR inhibitors range from hyperphosphatemia to ocular disorders like central serous retinopathy, and their overall efficacy and safety are still being tested in ongoing clinical trials [107]. However, they demonstrate powerful therapeutic efficacy, especially when coupled with ICIs.

## Conclusions

Current standards in BC rely on dated treatments such as platinum-based chemotherapies and BCG administration. Though these treatments are still being refined and improved, new treatment options are emerging. These new treatments utilize targeted routes to enhance the destruction of BC at its source. Many emerging therapies track certain molecular features commonly upregulated in metastatic BC cell lines. Therapies that target the upregulated PD-1/PD-L1 axis of BC cell lines allow clinicians to direct immune checkpoint-inhibiting treatments to the UC. ADCs afford clinicians the power to deliver cytotoxic drug payloads directly to the cells of choice in BC pathologies. Drugs that inhibit the well-understood PAM pathway can dysregulate the growth of metastatic BC cells. FGFR inhibitors have shown to increase survival rates in BC patients with FGFR2/FGF3 mutations, who have previously shown resistance to standard of care treatments. CAR-T cell therapies are still in their early preclinical state but show promise in the treatment of BC. A drawback of these emerging therapies is that many are still in preclinical or clinical trials, and their use has only been examined under well-controlled conditions and not in the general public. Importantly though, these therapies show that BC will soon be treated with more cell-specific targeting methods, allowing clinicians and patients greater control of treatment. Recent studies have shown that these therapies have a positive outlook for patient clinical outcomes, and their use in BC may be invaluable in the future.

## Abbreviations

ADC: antibody-drug conjugate
AKT: protein kinase B
BAK: Bacillus Calmette-Guerin-activated killer
BC: bladder cancer
BCG: Bacillus Calmette-Guerin
CAR: chimeric antigen receptor
CR: complete response
EGFR: epidermal growth factor receptor
EV: enfortumab vedotin
FGFR: fibroblast growth factor receptor
HER2: human epidermal growth factor receptor 2
ICIs: immune checkpoint inhibitors
IgG4: immunoglobulin G4
mAbs: monoclonal antibodies
mDOR: median duration of response
mTOR: mammalian target of rapamycin
mTORC1: mechanistic target of rapamycin complex 1
NK: natural killer
ORR: objective response rate
OS: overall survival
PAM: phosphoinositide 3-kinase/protein kinase B/mammalian target of rapamycin
PD-1: programmed cell death protein 1
PD-L1: programmed death ligand 1
PFS: progression-free survival
PI3K: phosphoinositide 3-kinase
SG: sacituzumab govitecan
T-DM1: trastuzumab emtansine
UC: urothelial carcinoma

## Declarations

### Copyright

© The Author(s) 2024.
